# Transgenic mice exhibiting inducible and spontaneous Cre activities driven by a bovine keratin 5 promoter that can be used for the conditional analysis of basal epithelial cells in multiple organs

**DOI:** 10.1186/1423-0127-16-2

**Published:** 2009-01-08

**Authors:** Chih-Chia Liang, Li-Ru You, Junn-Liang Chang, Ting-Fen Tsai, Chun-Ming Chen

**Affiliations:** 1 Department of Life Sciences and Institute of Genome Sciences, National Yang-Ming University, Taipei, Taiwan; 2 Institute of Biochemistry and Molecular Biology, National Yang-Ming University, Taipei, Taiwan; 3 Department of Pathology, Taoyuan Armed Forces General Hospital, Taoyuan County, Taiwan

## Abstract

**Background:**

Cre/*lox*P-mediated genetic modification is the most widely used conditional genetic approach used in the mouse. Engineered Cre and the mutated ligand-binding domain of estrogen receptor fusion recombinase (CreER^T^) allow temporal control of Cre activity.

**Results:**

In this study, we have generated two distinct transgenic mouse lines expressing CreER^T^, which show 4-hydroxytamoxifen (4-OHT)-inducible and spontaneous (4-OHT-independent) Cre activities, referred to *Tg(BK5-CreER*^*T*^*)I *and *Tg(BK5-CreER*^*T*^*)S*, respectively. The transgenic construct is driven by the bovine Keratin 5 promoter, which is active in the basal epithelial lineage of stratified and pseudo-stratified epithelium across multiple organs. Despite the difference in 4-OHT dependency, the *Tg(BK5-CreER*^*T*^*)I *and *Tg(BK5-CreER*^*T*^*)S *mouse lines shared similar Cre-mediated recombination among various organs, except for unique mammary epithelial Cre activity in *Tg(BK5-CreER*^*T*^*)S *females.

**Conclusion:**

These two new transgenic mouse lines for the analysis of basal epithelial function and for the genetic modification have been created allowing the identification of these cell lineages and analysis of their differentiation during embryogenesis, during perinatal development and in adult mice.

## Background

Gene targeting provides a powerful tool to address gene function by the manipulation of the mouse genome through homologous recombination in embryonic stem (ES) cells (reviewed in [1]). However, germ-line genetic modification often causes lethality or numerous effects that interfere with the analysis of specific biological phenotypes. Conditional gene targeting using the Cre/*loxP*-mediated recombination system (reviewed in [1,2]) offers an alternative approach for the dissection of gene function. Cre recombinase expression can be regulated by tissue or cell-type specific promoters in transgenic mouse lines. Thus, Cre can recognize *loxP *sites to catalyze site-specific recombination in a tissue/cell specific manner. In addition to tissue/cell specific regulation of Cre expression, temporal control of Cre recombinase activity in transgenic mice has been demonstrated utilizing Cre recombinase fused with the mutated hormone-binding domain of the estrogen receptor (ER^T^); this can be activated by the synthetic estrogen analog tamoxifen or 4-OHT, but not by the physiological ligand 17β-estradiol [3,4]. Thus, such an inducible Cre recombinase transgenic mouse model is able to further facilitate conditional gene knockout analysis and allow the study of gene function at specific time points in a highly controlled manner.

Keratin 5 (K5) is a member of type II keratins and expresses with its type I keratin partner keratin 14 (K14) in the basal layer of stratified squamous epithelium (SSC) [5-7]. Utilizing K5 promoter-driven reporter gene expression in transgenic mice has been shown to recapitulate the expression profiles of endogenous K5 in basal epithelia [8,9]; these cells are thought to have enriched stem/progenitor populations that give rise to the suprabasal differentiated cells of stratified epithelia [8-12]. Generation of transgenic mice expressing Cre recombinase driven by the K5 promoter as well as by the K14 promoter have provided very useful genetic tools for the analysis of the basal proliferating cells of SSC [13,14]. In addition, these reports have demonstrated that K5-Cre and K14-Cre mice exhibit Cre/*lox*P recombination activity through female germ-line only, which potentially confines the breeding strategy available for the analysis of tissue-specific gene ablation, that is in generalized germ-line deleted strains [13,14]. As an alternative, the K5 or K14 promoter directed Cre fused with either a mutated version of ER or PR (progesterone receptor) has allowed expression in a variety of transgenic mouse lines, which offers ligand-induced Cre/*lox*P-mediated recombination *in utero *or at adult stage; these have proved to be powerful genetic resources and have mostly concentrated on the analysis of epidermal development and disease [15-19]. To strengthen the genetic resources of the K5-derived epithelial lineages, we have generated transgenic mouse lines expressing the Cre recombinase fused with ER^T ^driven by the bovine K5 promoter on an inbred (C57BL/6J) background in this report.

## Methods

### Plasmid

**Figure 1:**
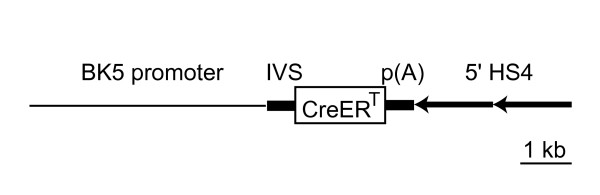
**Organization of transgenic construct *BK5-CreER*^*T *^for the production of *Tg(BK5-CreER^*T*^)S *and *Tg(BK5-CreER^*T*^)I *mice**. IVS, intron; p(A), SV40 polyadenylation; 5' HS4, insulator derived from the 5' boundary of the DNaseI hypersensitive site of the chicken β-globin locus.

The *BK5-CreER*^*T *^transgenic plasmid (Figure 1) was created by multiple subcloning steps and is composed of an excised 5.2-kb *Not*I-digested and *Nhe*I/Klenow filled-in bovine K5 promoter followed by an 0.5-kb intron sequence from the *BK5-Cre *plasmid (kindly provided by Dr. Richard R. Behringer with an agreement of Dr. José L. Jorcano), a 1.8-kb of *Eco*RI-digested/Klenow filled-in of *Cre-ER*^*T *^fusion gene derived from *pCre-ER(T) *plasmid (kindly provided by Dr. Richard R. Behringer with an agreement of Dr. Pierre Chambon), a 0.5-kb SV40 polyadenylation signal (pA) and 2 copies of the ~1.2-kb HS4 insulator sequence from the 5' region of the chicken β-globin locus (5' HS4; nucleotides 10~1199 from accession number U78775). This 5'-HS4 served as a barrier element that protects genes from any chromosomal position effect, which has been thought to result in histone modifications via USF proteins [20,21]. The 10.8-kb *Not*I/*Sal*I-digested transgene was eluted and separated from the *pBluscript *vector backbone for pronuclear microinjection [22].

### Mice

The *Tg(BK5-CreER*^*T*^*)I *and *Tg(BK5-CreER*^*T*^*)S *mice bearing the same transgene, *BK5-CreER*^*T *^(Figure 1) were generated by pronuclear microinjection and further maintained on a C57BL/6J background. ROSA26 Cre reporter mice (Gt(ROSA)26Sor^tm1Sor^; the Jackson Laboratory) on a C57BL/6;129 mix background are referred to as R26R mice in this report. The *Tg(BK5-CreER*^*T*^*)I/+;R26R/+ *and *Tg(BK5-CreER*^*T*^*)S/+;R26R/+ *bigenic mice were generated by crossing *R26R/R26R *females with *Tg(BK5-CreER*^*T*^*)I/+ *and *Tg(BK5-CreER*^*T*^*)S/+ *males, respectively. Occasionally, the offspring of *R26R/R26R *males and *Tg(BK5-CreER*^*T*^*)S/+ *females were generated and are referred to as *R26R;Tg(BK5-CreER*^*T*^)*S*, as they are the progeny of *Tg(BK5-CreER*^*T*^*)S/+ *females; these were used for the analysis of spontaneous Cre activity through the female germ-line. The genotyping protocol for the *R26R *allele was according to the methodology for PCR genotyping presented on the web site of the Jackson Laboratory. Genotyping of the *Tg(BK5-CreER*^*T*^*)I *and *Tg(BK5-CreER*^*T*^*)S *mice was determined by a 300-bp PCR product amplified by a forward primer (5'-GGACATGTTCAGGGATCGCCAGGCG-3') and a reverse primer (5'-CGACGATGAAGCATGTTTAGCTG-3'). The PCR conditions were 5 min at 94°C, 28 cycles for 1 min at 94°C, 1 min at 64°C, 1 min at 72°C and a final 2 min extension at 72°C. All animals were housed in microisolator cages (up to 5 mice per cage) using specific pathogen free husbandry. All experiments with mice were performed with the approval of the Institutional Animal Care and Use Committee (IACUC) at National Yang-Ming University.

### 4-OHT administration

4-hydroxytamoxifen (Sigma, St. Louis, MO, USA) was dissolved in dimethyl sulfoxide (DMSO; Sigma) at a concentration of 25 mg/ml. The 4-OHT solution was emulsified in sunflower seed oil (Sigma) by vortex followed by mixing on a rotator for 4~6 hours. The mice were injected intraperitoneally with three doses of 4-OHT (4~5 mg/kg body weight) every other day.

### Whole mount X-gal staining

The detailed experimental procedures had been described previously [23]. After X-gal staining, the tissues were embedded in paraffin and then were further processed to give ~7 μm thick sections. These tissue sections were placed on slides, deparaffinied and then rehydrated, which was followed by counterstaining with Nuclear Fast Red (Muto Pure Chemicals CO., Tokyo, Japan) for 10 minutes. β-galactosidase positive tissues were examined by light microscopy (BX51, Olympus, Japan).

## Results and discussion

Through pronuclear microinjection, four *BK5-CreER*^*T *^transgenic founders were obtained. Each transgenic founder was backcrossed to C57BL/6J for expansion of the individual transgenic line. All transgenic founders were fertile and transmitted *BK5-CreER*^*T *^transgenes to their progeny. To examine the Cre recombinase activity, *BK5-CreER*^*T *^mice were bred with *ROSA26 Cre *Reporter (*R26R*) mice [24] to generate *Tg(BK5-CreER*^*T*^*);R26R *bigenic mice; this was followed by intraperitoneal 4-OHT injection. Among these four transgenic mouse lines, three lines exhibited 4-OHT induced Cre recombinase activity. We selected an efficient and tightly controlled 4-OHT inducible *BK5-CreER*^*T *^transgenic mouse line, referred to here as *Tg(BK5-CreER*^*T*^*)I*, to be the representative line for further study.

**Figure 2:**
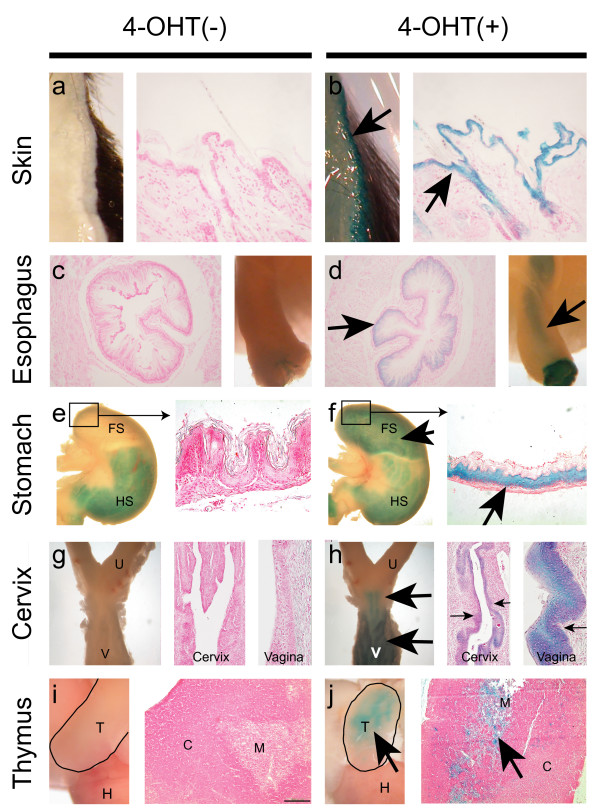
**Evaluation of 4OHT-induced Cre recombination in *Tg(BK5-CreER^*T*^)I;R26R *mice**. After 10 days of vehicle control (4OHT (-)) or 4OHT (+) treatment, the presence of β-galactosidase activity in the skin (a & b), the esophagus (c & d), the stomach (e & f), the lower female reproductive tract (g & h) and the thymus (i & j) was examined by X-gal staining. Histological sections of the corresponding X-gal stained organs were counterstained by Nuclear Fast Red and it was then possible to detect positive X-gal stained epithelial cells (arrows). Non-specific β-galactosidase activities were noticed in the hindstomach. FS, forestomach; HS, hindstomach; U, uterus; V, vagina; T, thymus; H, heart; M, thymic medulla; C, thymic cortex.

The 4-OHT inducible Cre activity of the *Tg(BK5-CreER*^*T*^*)I;R26R *bigenic mice was assessed in terms of reporter (*LacZ*) gene expression (Figure 2). We found that all K5 expressing tissues including the epidermis and its derivatives (Figure 2b), the esophageal (Figure 2d) and the foregastric mucosa (Figure 2f), the cervicovaginal epithelia of the female reproductive tract (Figure 2h) as well as the medullary thymic epithelium (mTECs; Figure 2j) showed β-galatosidase activity when analyzed by whole-mount X-gal staining following histological sectioning. In general, X-gal positively staining of the stratified and pseudo-stratified epithelia across multiple organs was noticed. Therefore, our results are complementary to previous reports that generated *K5-CreER*^*T *^and *K5-CreER*^*T*2 ^transgenic mice and mainly concentrated on epidermal Cre-mediated recombination [18,19].

**Figure 3:**
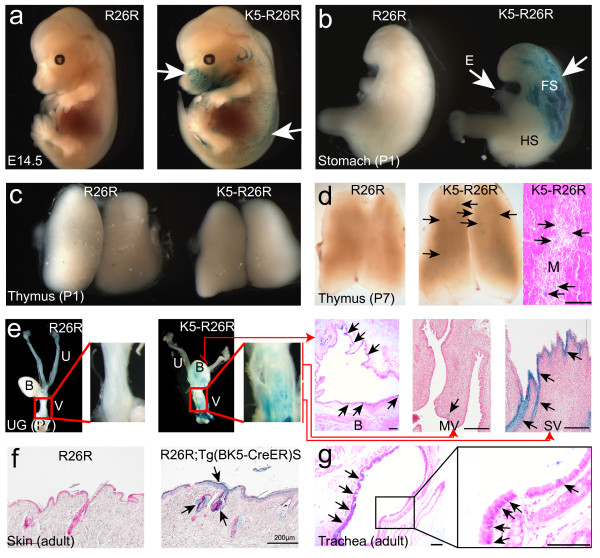
**Evaluation of spontaneous Cre recombination in *Tg(BK5-CreER^T^)S;R26R *mice**. a: E14.5 embryos (left panel, *R26R/+ *(*R26R*) control; right panel, *Tg(BK5-CreER*^*T*^*)S/+;R26R/+ *abbreviated as *K5-R26R*) were stained for β-galactosidase activity, which appeared in whisker and hair follicles of the *K5-R26R *embryo (arrows). b: Dissected stomach from newborn mice (P1) after whole mount X-gal staining revealed the presence of intensive blue-stained areas in the esophagus (E) and forestomach (FS; arrows) of *K5-R26R *mice. c & d: Dissected thymus from *R26R *or *K5-R26R *mice underwent whole mount X-gal staining and histological section and this revealed scattering blue foci (arrows) in the *K5-R26R *thymic medulla (M) at P7 (d) but not at P1 (c). e: The urogenital system of *R26R *or *K5-R26R *females at P7 was dissected and stained for β-galactiosidase activity. Histological sections of corresponding X-gal stained organs of a *K5-R26R *female are shown in the right panels. Arrows indicate positively stained epithelium; B, bladder; U, uterus; V, vagina; SV, sinus vagina; MV, Müllerian vagina; f & g: Histological sections of X-gal stained skin and trachea from adult offspring (*R26R *and *R26R;Tg(BK5-CreER*^*T*^*)S*) obtained by crossing a *R26R/R26R *male with a *Tg(BK5-CreER*^*T*^*)S *female. The β-galactosidase activity showed tissue/cell-specific expression in the epidermal, hair follicle and sebaceous cells of the *R26R;Tg(BK5-CreER*^*T*^*)S *skin (arrows in f) and in the basal cells of *R26R;Tg(BK5-CreER*^*T*^*)S *trachea (arrows in g). Scale bars, 100 μm.

Interestingly, one mouse line exhibited spontaneous Cre recombinase activity in the absence of 4-OHT induction and this line is referred to as *Tg(BK5-CreER*^*T*^*)S*. The detailed mechanism that triggers this spontaneous Cre activity remained unclear. X-gal stained tissues of *Tg(BK5-CreER*^*T*^*)S;R26R *bigenic mice revealed that Cre activity initiated reporter LacZ gene expression started at around embryonic day (E) 14.5 (Figure 3a). Histological sections revealed that X-gal positive cells appeared in the epidermis, the whisker follicles, the hair follicles (Figure 3a), the nasal cavity and the oral cavity (data not shown). At birth, the esophagus and the forestomach, but not in the hindstomach (HS), also showed strong X-gal stained patterns (Figure 3b). At this stage, the mTECs were expected to exhibit β-galactosidase activity because previous reports showing that the thymic epithelial progenitors of developing thymus indicated expression by both K5 and K8 at E12.5 [25], while adult mTECs expressed K5 specifically [26]. However, the spontaneous β-galactosidase activity in the thymic epithelium of *Tg(BK5-CreER*^*T*^*)S;R26R *mice was undetectable at birth (Figure 3c) and was only detected as showing a scattered pattern until postnatal day (P) 7 (Figure 3d). Thus, our findings suggested that the expression timing of CreER^T ^in some organs, such as the thymus, of *Tg(BK5-CreER*^*T*^*)S *mice might not exactly match that of endogenous K5 expression during organogenesis. Furthermore, in the urogenital system of the *Tg(BK5-CreER*^*T*^*)S;R26R *females at P7, the transitional epithelia lining of the bladder and the stratified epithelia lining of sinus vagina (SV) markedly exhibited β-galactosidase activity, whereas very few X-gal positively stained epithelial cells in the Müllerian vagina (MV) could be detected (Figure 3e). The differential X-gal positive staining in the MV compared to SV was possibly due to the more advanced epithelial stratification of SV compared to MV, which is initially lined by simple columnar epithelia that resembles the uterine epithelia, and then transits to stratified epithelia at perinatal stage [27,28].

Previously Hafner et al and Ramirez et al. independently demonstrated that K5-Cre and K14-Cre express in female oocytes and have suggested their applicability as female germ-line deleter strains [13,14]. To determine whether our *Tg(BK5-CreER*^*T*^*)S *transgenic mice exhibited female germ-line Cre activity, we obtained offspring (*R26R;Tg(BK5-CreER*^*T*^*)S*) from a cross of a *R26R/R26R *male and a *Tg(BK5-CreER*^*T*^*)S/+ *female and analyzed spontaneous β-galactiosidase expression compared to of control *R26R *mice. Our results showed that the X-gal positively stained patterns remained specific to the K5-expressing cell types as shown by examples of skin and trachea (Figure 3f &3g). In the *R26R;Tg(BK5-CreER*^*T*^*)S *skin, the β-galactosidase activity showed tissue/lineage-specific expression in the epidermal, hair follicles and sebaceous cells (Figure 3f), suggesting that the stem cell populations were targeted. In the *R26R;Tg(BK5-CreER*^*T*^*)S *trachea, intense X-gal-stained cells were found in the basal cells of the pseudo-stratified epithelium (Figure 3g). Our data suggested that the *Tg(BK5-CreER*^*T*^*)S *mouse line does not behave as a female germ-line deleter as described in earlier reports [13,14]. The spontaneous Cre activity of *Tg(BK5-CreER*^*T*^*)S *mice enabled us to bypass the female germ-line deletion effect and to use this line for Cre-mediate recombination in a tissue-specific manner during embryonic and early postnatal development.

**Figure 4:**
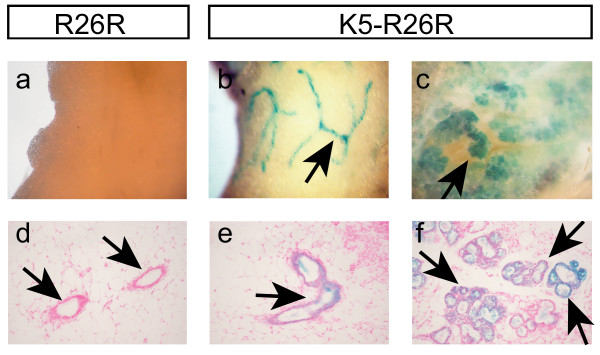
**Analysis of spontaneous Cre recombination in *Tg(BK5-CreER^*T*^)S;R26R *mammary glands**. a-c: Whole mount X-gal staining of mammary fat pads; Arrows indicate ductal and alveolar structures that show X-gal positive staining in the *K5-R26R *mammary glands of virgin (b) and lactation (c) stage mice; this staining is not present in R26R mice (a). d-f: Histological sections of the corresponding X-gal stained mammary glands shown in a-c. Arrows indicated X-gal stained basal and luminal epithelial cells. a-c, cropped original magnification ×8; d-f original magnification ×200.

**Figure 5:**
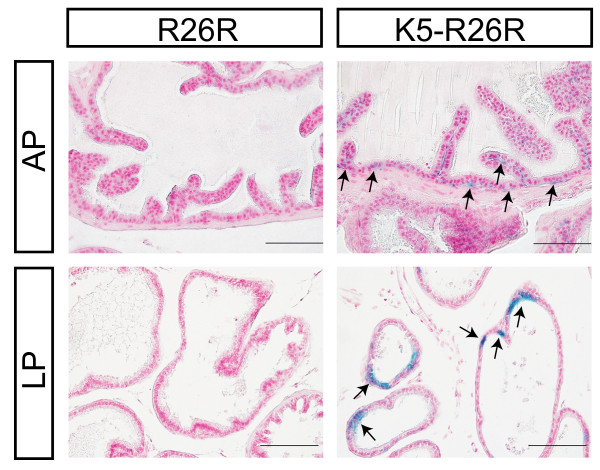
**Analysis of spontaneous Cre activity in *Tg(BK5-CreER^*T*^)S;R26R *prostate glands**. Histological sections of anterior and lateral prostate lobes (AP & LP) from control R26R/+ and *Tg(BK5-CreER*^*T*^*)S;R26R *(K5-R26R), which were counterstained by Nuclear Fast Red. The results revealed X-gal positively stained epithelial cells (arrows). Scale bars, 100 μm.

Several organs such as mammary and prostate glands are not typically considered to be stratified epithelium, but these epithelial glands are composed of K5 expressing basal epithelial cells and terminal differentiated luminal epithelium. In our *Tg(BK5-CreER*^*T*^*)S *transgenic mice, but not in our *Tg(BK5-CreER*^*T*^*)I *transgenic mice, Cre-mediated recombination in the mammary gland was detected and X-gal-stained cells were observed in both the luminal epithelium and the basal epithelial cells of the mammary glands at P7 (data not shown), in virgins (Figure 4b &4e) and also during lactation (Figure 4c &4f). In addition, prostate basal cells expressing K5 that showed β-galactosidase activity were found in the basal compartment of the *Tg(BK5-CreER*^*T*^*)S;R26R *anterior prostate (AP) lobes (Figure 5); however, this contrasted with the fact that expression was present in both the basal and the luminal epithelium of the lateral prostate (LP) lobes (Figure 5). These findings indicated that Cre activity can be present in a subpopulation of epithelial glands and will enabled us to define molecular and cellular mechanisms precisely using these mouse lines within a subpopulation using lineage tracing or conditional genetic experiments.

Taken together, we reported two new transgenic mouse lines, *Tg(BK5-CreER*^*T*^*)S *and *Tg(BK5-CreER*^*T*^*)I*, which exhibit spontaneous and 4-OHT-inducible Cre activity driven by a bovine K5 promoter, respectively. For analysis of Cre/*lox*P-mediated recombination at the embryonic and early postnatal stages using *Tg(BK5-CreER*^*T*^*)S *mouse line, a breeding scheme that considers whether the male or the female is carrying the *BK5-CreER*^*T *^transgene is not necessary because there was no female germ-line Cre-mediated recombination in our *Tg(BK5-CreER*^*T*^*)S *females. The 4OHT-induced *Tg(BK5-CreER*^*T*^*)I *mouse line will enable us to perform conditional genetics in temporally and spatially controlled manner.

## Conclusion

These genetic resources generated in this study will not only help us to understand the biology of the skin, but will also support studies of the basal epithelial lineages of pseudo-stratified, stratified and transitional epithelia as they appear in the thymic medulla, the esophagus, the forestomach, the trachea, the bronchus, the bladder, the cervicovagina, etc. Moreover, glandular organs such as prostate glands, which are composed of K5-expressing basal epithelium, can also be a target organ using these new transgenic mouse lines; this will allow the genetic fine-dissection of basal epithelial function in terms of epithelial identity and differentiation.

## Competing interests

The authors declare that they have no competing interests.

## Authors' contributions

CCL performed the experiments and analyzed the data. LRY and JLC participated in the data interpretation. TFT contributed the materials for the initial design of transgenic construct. CMC performed the experiments, analyzed the data and wrote the manuscript. All authors read and approved the final manuscript.
